# Nanophotonics: Energy Transfer towards Enhanced Luminescent Chemosensing

**DOI:** 10.3390/ma8041682

**Published:** 2015-04-13

**Authors:** Roy Aad, Christophe Couteau, Gilles Lérondel

**Affiliations:** Laboratoire de Nanotechnologie et d’Instrumentation Optique, Institut Charles Delaunay, CNRS UMR 6281, Université de Technologie de Troyes, 12 rue Marie Curie, BP 2060, 10010 Troyes Cedex, France; E-Mail: christophe.couteau@utt.fr

**Keywords:** luminescence, chemosensing, nanophotonics, energy transfer, zinc oxide

## Abstract

We discuss a recently proposed novel photonic approach for enhancing the fluorescence of extremely thin chemosensing polymer layers. We present theoretical and experimental results demonstrating the concept of gain-assisted waveguided energy transfer (G-WET) on a very thin polymer nanolayer spincoated on an active ZnO thin film. The G-WET approach is shown to result in an 8-fold increase in polymer fluorescence. We then extend the G-WET concept to nanostructured media. The benefits of using active nanostructured substrates on the sensitivity and fluorescence of chemosensing polymers are discussed. Preliminary theoretical results on enlarged sensing surface and photonic band-gap are presented.

## 1. Introduction

With the rising illicit use of improvised explosive devices, explosives trace detection has become a major societal, governmental and military concern. One of the major challenges in explosives trace detection lies in the low vapor concentrations that are generated by explosive materials. For instance, trinitrotoluene TNT exhibits a saturated vapor concentration of 5–10 parts per billion (ppb) at 25 °C; while new explosive materials, such as the HMX, can exhibit a saturated vapor concentration lower than 1 part per quadrillion [1]. In the head space over the explosive, the trace concentrations are of course orders of magnitude lower than the saturated vapor concentration.

Over the past few years, various analytical techniques have been investigated in order to develop operational explosives sensing devices [2,3]. Between the various analytical techniques, Ion Mobility Spectrometry (IMS) has gained a universal acceptance as an efficient technique for explosives detection, with more than 10,000 IMS devises in use in airports worldwide [4]. Nonetheless, analytical techniques stayed rather bulky and expensive and therefore non-suitable for amenable on-site explosives detection.

Recent research studies have concentrated on the concept of “chemosensors” as a solution to achieve sensitive, selective, portable and non-expensive explosives trace detectors. Chemosensors are transducing materials that are able to change one or more of their physical properties in the presence of certain chemical elements in the environment. Since explosives are known to be electron-poor materials, most of the explosives chemosensors work on the principle of electron exchange from the chemosensor towards the explosive substance. Most of the explosives chemosensing setups can be thus summarized into two main approaches. The first approach consists on the use of conductive materials, such as graphene sheets or carbon nanotubes [5,6,7]. Explosives trace detection, in this case, is done by monitoring changes in resistivity/conductivity of the chemosensing material, which is therefore usually called “chemiresistors”. While chemiresistors can exhibit limits of detection (LOD) down to a few ppb; they however lack selectivity which is essential for sensing applications. The second approach consists on the use of fluorescent polymers. Explosives trace detection, in this case, is realized through a fluorescence “quenching” process, *i.e.*, a decrease in the fluorescence intensity, [8] which occurs in the presence of explosive analyte. The approach was first proposed by Tim Swager’s group in the late 90 s [9,10], where the group investigated the sensitivity of polyphenyl ether (PPE) based conjugated polymers thin films towards TNT vapor. For a 2.5 nm thick polymer film, the group observed a 70% fluorescence quenching at 60 s of exposure to TNT. This sensitivity is however strongly related to the polymer thickness. Swager’s group showed that, due to strong polymer-analyte (*i.e.*, slow analyte diffusion), the polymer films can be quenched with only surface-bound analyte. For 20 nm thick polymer film, the group observed only a 30% fluorescence quenching at 60 s of exposure to TNT. In 2002, Swager’s group further enhanced the fluorescence quenching properties of PPE-based polymers through the synthesis of a triphenylene-based PPE [11,12]. In its aggregate solid state, the triphenylene-based PPE formed a chiral grid, which resulted in longer excited-state lifetime, higher quantum yield and improved exciton diffusion length. Under similar experimental conditions as [9], the triphenylene-based PPE thin films showed a 4-fold increase in sensitivity towards TNT vapor (75% fluorescence quenching within 10 s). In 2008, E. Obert investigated on polysiloxane-based polymer for explosives detection. The polysiloxilane backbone has faster analyte diffusion due to higher polysiloxylane permeability to gases. Obert compared the sensitivity of various polysiloxane-based and PPE-based polymers thin films. The various polymer thin films had a thickness of 10 nm and were exposed to 2,4-DNT for 60 s. Obert concluded that the polysiloxylane-based polymers presented higher quantum yield and quenching efficiency compared to PPE-based polymers [13]. Swager’s group and Ober’s work did not however address the issue of selectivity. In 2012, Che *et al.* reported on TNT detection using a highly selective and highly sensitive carbazole-based tetracycle [14]. In its aggregate solid state, the carbazole-based tetracycle formed a nanoporous thin film allowing for a diffusion-controlled detection of TNT. Moreover, through the introduction of a carboxyl group, Che *et al.* were able to match the energy of the HOMO state of the polymer to that of the LUMO state of TNT. The polymer exhibited a strong quenching (75% fluorescence quenching within 10 s) when exposed to TNT. However, the polymer presented no fluorescence quenching when exposed to other oxidizing reagent vapors.

Due to their interesting sensing properties, fluorescence sensing polymers (FSPs) have, thus far, emerged as the most promising chemosensing materials for explosives trace detection applications. Nevertheless, polymers LOD are still to be improved in order to achieve operational use. It is essential to stress that the results reported above are all obtained at saturated vapor concentrations. On the other hand, the use of extremely thin FSP layers is a necessary condition in order to achieve low LOD. Extremely thin layers assure homogeneous analyte diffusion throughout the layer thickness which in turn assures high quenching efficiencies in the FSP layer [9]. Therefore, most of the FSP-based chemosensing results were reported on extremely thin polymer layers, which do not exceed a thickness of 10 nm. However, the decrease of the FSP layer thickness is accompanied by a drastic decrease of the optical excitation and number of emitters. As a result, extremely thin layers exhibit a poor fluorescence signal that limits the LOD of the sensing layer. Hence, the increase of the fluorescence signal of extremely thin FSP layers is a major challenge for achieving the required LOD for explosives trace detection.

In this article, we present a research summary on a novel and global photonic concept of gain assisted waveguiding energy transfer (G-WET) for enhancing the fluorescence of extremely thin FSP layers. The theoretical and experimental results presented herein reveal the potential of the G-WET proposed concept, which is also of interest for various sensing, lighting and photovoltaic applications. An extension of the G-WET concept towards more complex photonic structure is discussed later on.

## 2. Materials and Concept

### 2.1. The Concept

**Figure 1:**
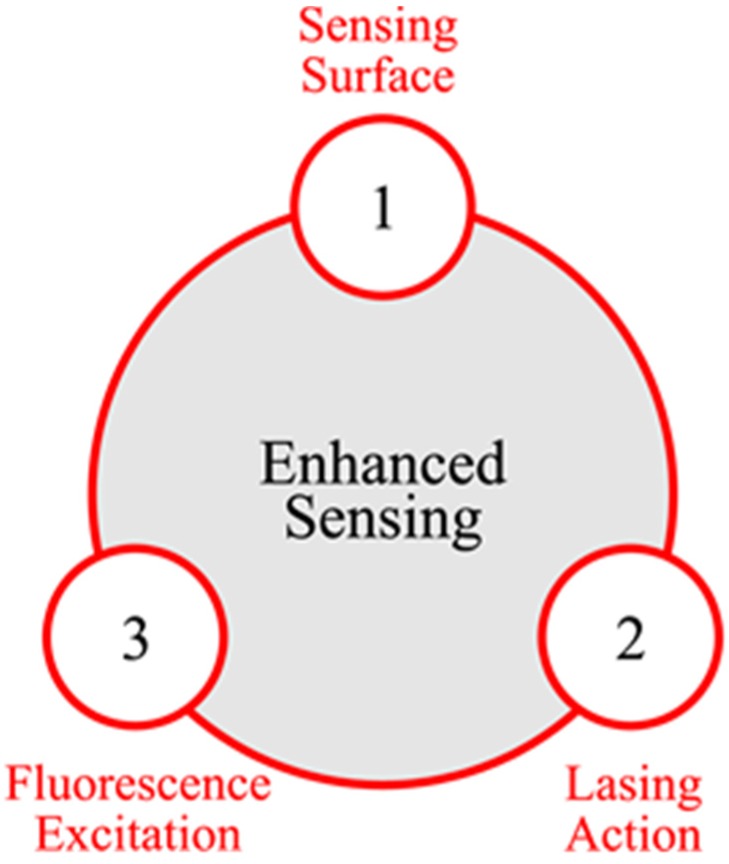
Synoptic illustration of the three main approaches that can be considered for enhancing the limits of detection of fluorescent sensing polymers.

Figure 1 presents a synoptic illustration of the 3 main approaches for enhancing the limits of detection of fluorescent sensing polymers.

The first approach consists on enlarging the sensing surface which is defined by the area of the polymer surface in contact with the ambient medium (in our case the air). The approach is purely geometrical and has the merit of being simple. The sensing surface can be easily enlarged by coating the polymer layer on non-planar surfaces. The enlarged sensing surface leads to gain in sensitivity due to the increased probability of analyte adsorption [15]. The approach was tackled by Zhu *et al.* whom showed that coating a FSP on arrays of aligned ZnO nanorods allowed for better response time and quenching efficiency while having higher polymer fluorescence when compared to a polymer layer coated on a standard quartz plate [16]. The enhancement reported by Zhu *et al.* was purely geometrical as the samples were optically pumped at 400 nm, therefore only exciting the polymer layer.

The second approach consists on enhancing the polymer emission via lasing action. Lasing action is known to drastically increase the internal quantum efficiency (*i.e.*, number of emitted photons) in luminescent films. Lasing action is usually manifested by a super-linear increase in luminescence intensity with increasing pumping intensities. Nevertheless, lasing threshold, and consequently lasing action, is strongly dependent to optical losses within the luminescent layer. As a matter of fact, the lasing threshold increases with increasing optical losses. Optical losses, induced by analyte adsorption, can therefore lead to an increase in the polymer lasing threshold. Due to the super-linear lasing action dependence, the slightest increase in the optical losses results in a drastic (super-linear) decrease in the polymer fluorescence. Lasing action can thus leads to important gain in polymer sensitivity. The approach was addressed by Rose *et al.* whom proposed a new polymer structure which exhibited amplified spontaneous emission (ASE). The authors demonstrated important gains in polymer sensitivity by working near the lasing threshold of the polymer ASE [17]. The approach however is strictly limited to the polymer structure and its optical properties.

The third approach consists on enhancing the polymer fluorescence excitation. The approach has never been addressed before, until recently by our team [18,19], and forms the main subject of this article.

**Figure 2:**
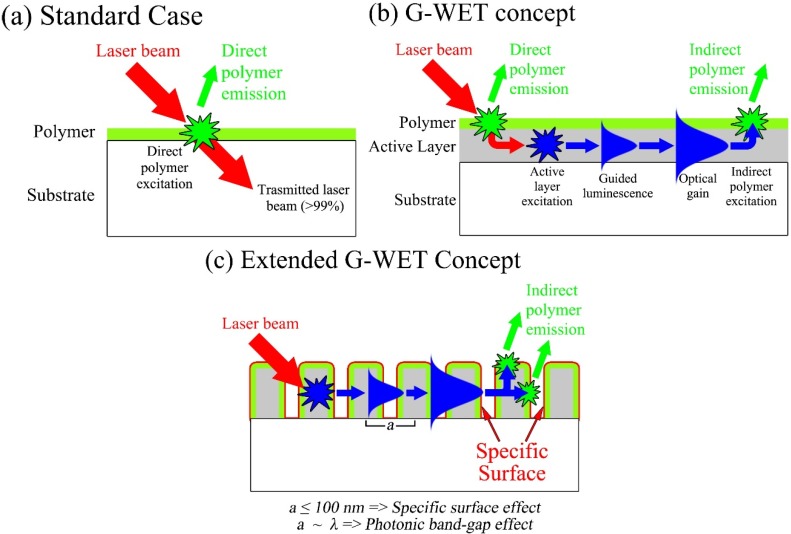
(**a**) A 2D illustration of the standard case of a fluorescent polymer layer on passive substrate; (**b**) A 2D illustration of the gain-assisted waveguided energy transfer (G-WET) concept. The 2D illustration depicts a fluorescent polymer layer on active layer along with the various phases leading up to the G-WET; (**c**) A 2D illustration of the extended G-WET concept. The 2D illustration depicts a fluorescent polymer layer on a nanostructured active layer. Both specific surface and photonic effect can be considered.

Figure 2a,b present 2D illustrations which clarify the fluorescence excitation problematic and the proposed concept. Figure 2a depicts the standard case of an extremely thin polymer layer on passive substrate. In most chemosensing setups, the polymer layer is typically coated on quartz substrate. In such case, most of the laser excitation (>99%) is transmitted through the polymer layer and absorbed in the quartz substrate. Only an extremely small amount of the laser excitation serves to excite the polymer fluorescence. The used of a passive substrate thus leads to an inefficient fluorescence excitation.

Figure 2b on the other hand presents the G-WET concept for a planar geometry. Figure 2b depicts the case of an extremely thin polymer layer coated on an active (*i.e.*, luminescent) waveguiding slab. Similarly to Figure 2a, most of the laser excitation is transmitted through the polymer layer and only a small amount serves to excite the polymer. For Figure 2b however, the laser excitation transmitted through the polymer is absorbed in the active layer and excites the active layer luminescence. The waveguiding configuration of the active layer insures that the layer luminescence is majorly coupled into the guided mode. The guided mode (represented by the Gaussian profile in Figure 2b) stays confined within the active layer and excites the polymer layer via the tail of its evanescent wave. Guided modes thus provide the most efficient configuration to excite the fluorescence of extremely thin polymer layers. Gain in the active layer helps to further increase the guided mode intensity, as shown in Figure 2b, leading to a more efficient polymer excitation. It is important to stress that guided modes can travel on long distances (couple of mm) inside the active layer. Guided modes can therefore excite a larger area of the polymer layer, exceeding the area defined by the laser spot. Guided modes are therefore at the basis of a ‘Geometrical effect’ that is manifested by an enlarged fluorescent surface.

Spin-coating the polymer layer on an active waveguiding slab thus enhances the polymer fluorescence excitation through the absorption and emission processes occurring within the slab layer. Nonetheless, a number of criteria must be verified to achieve an efficient G-WET effect, which are: the active layer must exhibit high luminescence intensity (ideally stimulated emission with gain), a high refractive index, and a luminescence peak that is within the polymer absorption range. The role of each criterion is discussed in detail in [19].

While it is clear that waveguiding slabs lack optical modulation properties that can be found in nanostructured materials; they however present interesting physical and photonic properties. The planar geometry of waveguiding slabs provides rigidity and ease of process. For instance, the growth of dielectric thin films is a well mastered process which can be done through a variety of controlled growth techniques in order to obtain the desired thin film crystal quality and thickness. Moreover, the smooth surface of the planar geometry allows for a high uniformity of the polymer layer (*i.e.*, the sensor), which can be easily processed by spin-coating techniques.

In an ultimate approach, one can imagine to combine the G-WET effect to the solutions proposed by Rose *et al.* and Zhu *et al.* [16,17].

Figure 2c presents a 2D illustration of the extended G-WET concept. Figure 2c depicts an extremely thin polymer layer coated on a nanostructured active layer. Nanostructured materials present many interesting properties that can be beneficial for sensing applications. For instance, densely stacked nanostructures, with an extremely small lattice parameter to emission wavelength (*i.e.*, *a* << λ), present high specific surfaces which can lead to an important enhancement in the polymer sensing surface. On the other hand, nanostructures with a lattice parameter that is comparable to the emission wavelength (*i.e.*, *a* ~ λ) present interesting optical modulation properties. For instance, such nanostructures can exhibit optical feedback properties that could help decrease lasing threshold and thus reach more efficient lasing action [20,21].

Coating the polymer layer on a nanostructured active layer can thus lead to an important enhancement in polymer sensitivity resulting simultaneously from the enlarged sensing surface, the optical modulation, and G-WET. However, sensing surface, optical modulation, and G-WET properties of the structure are tightly linked to the structure parameters. G-WET is mainly insured by the optical properties of the active substrate [19]. However, a fine tuning of the structure parameters is essential in order to maintain the waveguiding configuration that is crucial for G-WET. Similarly, specific surface, which is purely a geometrical effect, and optical feedback, which results from the refractive index modulation, are defined by the structure parameters. However, sensing surface, optical modulation, and G-WET properties do not behave similarly and, therefore, present optimum values for differing structure parameters. Therefore, the optimum structure parameters can only be the result of a compromise between the three considered effects. However, such a compromise requires a profound knowledge of the weight of each effect on polymer fluorescence. The determination of optimum structure parameters is thus a major theoretical and experimental challenge.

In addition to modeling challenges, nanostructured substrates present several processing difficulties. Nanostructuring often requires the application of complex bottom-up and top-down techniques, therefore increasing production time and cost. Moreover, nanostructured substrates can induce non-uniformity in the sensor, which requires an optimization of the polymer coating process.

Herein, we present theoretical and experimental validation of the G-WET concept for planar geometries. Concerning the extended G-WET concept, we restrict our study to preliminary results on the enlarged sensing surface and photonic properties of the nanostructures.

### 2.2. Studied Polymer

To validate the concept, we consider the case of poly[dimethyl-co-methyl-(1,1,1-trifluoro-2-(trifluoromethyl)-2-oxy-pent-4-yl)-co-(methyl-(4-pentyloxy-(N-(2,5-di-tert-butylphenyl))-1,8-naphthalimide)]-siloxane [22]. The polymer structure consists of a (non-emissive) polysiloxane backbone to which is introduced a fluorescent 1,8-naphthalimide moiety. The naphtalimide moiety presents a maximum absorption wavelength at 365 nm and a maximum emission wavelength at 468 nm. As reported in [22], the naphtalimide fluorescence is quenched in the presence of nitroaromatic compounds, such as DNT and TNT, due to the electron transfer between the electron-rich naphtalimide and the electron-poor nitroaromatic compound. The polymer synthesis process is decribed in detail in [19].

### 2.3. Zinc Oxide

ZnO is largely investigated as a low-cost effective replacement for ultraviolet (UV) emitting materials, such as gallium nitride (GaN), due to its direct-wide-gap (~3.3 eV) and large exciton binding energy (~60 meV), typically 2.4 times higher than room-temperature thermal energy (~25 meV), which allows for intense excitonic near-band-edge emission and laser action at room temperature. Under low pumping intensities, defect-free ZnO thin films exhibit a free-exciton emission peak, denoted *E*_ex_, at a wavelength of 375 nm (3.3 eV) that grows linearly with pumping intensity [23]. Moreover, room temperature stimulated UV emission can occur in high quality ZnO thin films at high pumping intensities [24]. The stimulated ZnO emission is characterized by the rapid appearance of sharp and red-shifted emission peaks, displaying a super linear increase with pumping intensity [23].

ZnO thin films can exhibit two distinct stimulated emission peaks, denoted *P* and *N*. The *P*-line is generally attributed to an exciton-exciton collision process in which an exciton recombines to generate a photon after transferring, by inelastic collision, part of its energy to another exciton that is scattered to a continuum of states. The *P*-line exhibits a fixed red-shift of the peak energy and an 8th power PL dependence on pumping intensity. On the other hand, the *N*-line is associated to radiative recombination of electron-hole plasma (EHP). EHP is observed at extremely high pumping intensities, capable of generating carrier densities which exceed the Mott transition density, therefore resulting in a dissociation of the excitons due to strong coulomb interactions. The *N*-line is characterized by a non-constant red-shift of the peak energy and a 5th power PL dependence [23]. Stimulated emission is normally associated to optical gain. Various studies reported on optical gains up to 1369 cm^−1^ for the *P* and *N* emission [23,25,26,27,28,29].

The high refractive index is another interesting feature of ZnO. Ellipsometry studies on ZnO thin films reported a real part refractive index ranging between 2 in the visible to 2.5 in the UV [30,31]. The refractive index of sapphire is usually around 1.8. Thus, a ZnO thin film grown on sapphire is a dielectric planar waveguide capable of supporting guided modes. The waveguiding configuration of ZnO thin films is a keystone feature for stimulated emission, as it insures the optical feedback necessary for lasing. This is further demonstrated by the disappearance of the stimulated emission in ZnO thin films with thicknesses below the cut-off [25].

ZnO is therefore chosen as the active material as it verifies all the criteria that are required to have an efficient G-WET effect. ZnO thin films were grown on sapphire substrates in order to realize the experimental study. The growth process by which the ZnO thin films were obtained is detailed in [19]. It is important to stress that most of the fluorescent polymers which are used for explosive chemosensing applications are excited by UV-blue light [9,10,11,12,13,14,15,16,22]. Thus, ZnO is not only adequate for exciting the FSP studied herein, but also adequate for most of the studied FSPs. The use of ZnO thin films and nanostructures therefore presents a general photonic solution for the enhanced sensing issue.

## 3. Results and Discussion

Theoretical modeling is essential prior to any experimental investigation, in order to fully understand the various physical phenomena at play and optimize structure parameters. The theoretical modeling, presented herein, considers the case of an extremely thin (5 nm) FSP layer coated on a ZnO thin film grown on a sapphire substrate. The Source-Terms method is applied in order to model the emission of the FSP layer and ZnO thin film [18,32]. The applied model was shown to present good agreement with experimental results [33].

**Figure 3:**
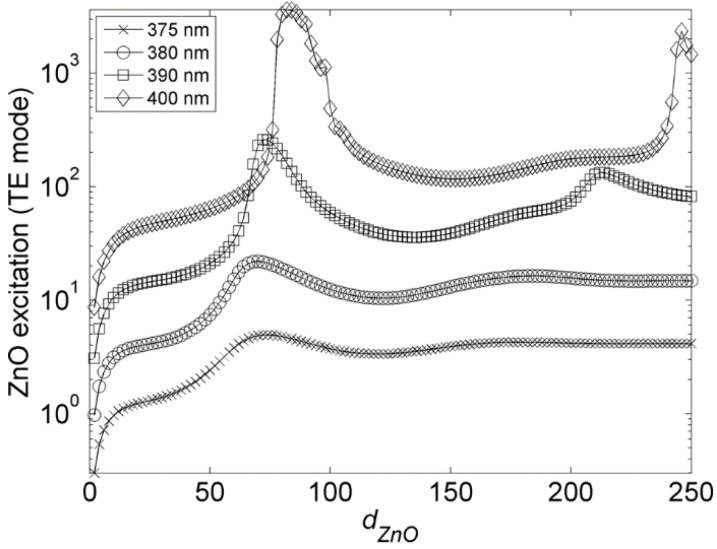
Evolution of the ZnO excitation as a function of the ZnO layer thickness (*d*_ZnO_) and emission wavelength. Graphs are shifted by half an order of magnitude and normalized to *d*_ZnO_ = 0. Reprinted with permission from [18]. Copyright 2010 AIP Publishing LLC.

Figure 3 presents the evolution of the ZnO excitation of the FSP layer (*i.e.*, the intensity of the ZnO emission inside the FSP layer) as a function of the ZnO film thickness (*d*_ZnO_). The graphs presented in Figure 3 are normalized to *d*_ZnO_ = 0 and shifted by half an order of magnitude in the *y*-axis. Since ZnO emission is preferably TE-polarized [33,34], only the TE mode was considered in the calculations. Calculations were carried out at the wavelengths of 375 nm (×), 380 nm (○), 390 nm (□), and 400 nm (◊), in order to account for the spectral redshift which occurs in ZnO emission when it passes from a spontaneous emission regime (at 375 nm) to a stimulated emission regime (*cf.* Section 2.3).

Figure 3 reveals that a ZnO excitation optimum exists for each of the considered wavelengths. Optimum ZnO excitation occurs for thicknesses of *d*_ZnO_ = λ_ZnO_/2*n*_ZnO_, where λ_ZnO_ and *n*_ZnO_ are respectively the emission wavelength and refractive index of the ZnO thin film. At these thicknesses, the ZnO thin film is a single-mode waveguiding slab. Figure 3 shows that the gradual redshift of the ZnO emission from 375 nm to 400 nm results in a drastic increase in the ZnO excitation. This is due to the emphasis of the guided mode [18], as the extinction coefficient (κ) is reduced from 0.311 at 375 nm to 0.004 at 400 nm [30].

Figure 3 shows that guided ZnO emission has more important impact on the FSP layer excitation compared to the outwards-emitted ZnO emission. ZnO guided emission provides the most efficient way to excite the FSP layer, as it allows for the FSP layer to be repeatedly excited via the tail of the evanescent wave.

In order to experimentally validate the G-WET, extremely thin (10 nm) FSP (*cf.* Section 2.2) layers were spincoated on a quartz substrate (sample denoted S0) and a 170 nm thick ZnO thin film grown on sapphire (sample denoted S1) [19]. The samples were excited using a pulsed nitrogen laser (λ = 337.1 nm, 4 ns pulse duration) operating at a repetition rate of 10 Hz. Polymer fluorescence was collected using an objective lens with a 0.13 numerical aperture focused onto a large core (400 µm diameter) optical fiber connected to a 50 cm focal length spectrometer equipped with a CCD Peltier-cooled camera. Neutral density filters were used in order to control the pumping intensity.

**Figure 4:**
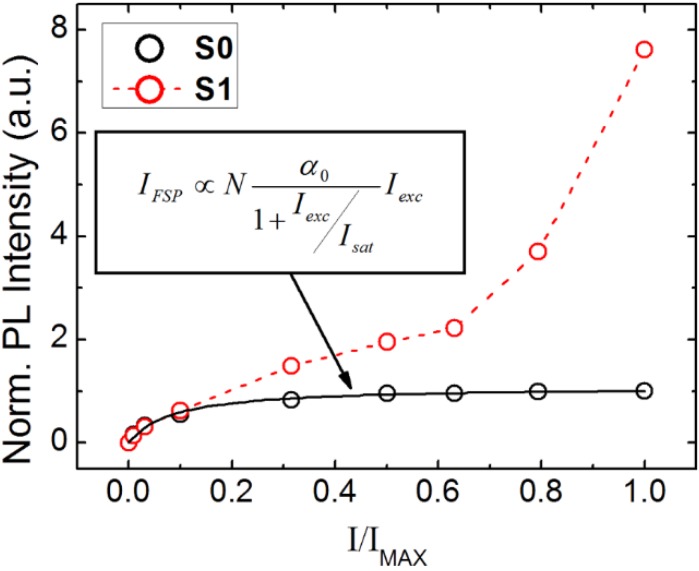
Comparison between the photoluminescence of the fluorescent polymer film coated on quartz, S0 sample (black circles) and on the ZnO layer, S1 sample (green circles) as a function of the normalized pumping intensity (*I*/*I*_MAX_). The plotted PL intensity values are taken at λ ~ 468 nm and normalized by the maximum PL intensity recorded for the S0 sample. Reprinted with permission from [19]. Copyright 2014 American Chemical Society.

Figure 4 presents the evolution of the FSP peak intensity (λ = 468 nm) for S0 and S1 samples as a function of the normalized pumping intensity (*I*/*I*_MAX_). Peak intensities are normalized to the maximum peak intensity presented by S0 (*i.e.*, at *I*/*I*_MAX_ = 1). Figure 4 shows that the FSP layer coated on quartz (*i.e.*, S0) saturates in the fluorescence peak intensity (black circles) at high pumping intensities according to the formula in insert. The fluorescence saturation of S0 is attributed to the absorption saturation of the FSP layer lying under direct laser excitation [19].

On the other hand, Figure 4 reveals that the FSP layer coated on ZnO (*i.e.*, S1) exhibits a superlinear (2.5 power) increase in fluorescence peak intensity (red circles) at high pumping intensities, eventually reaching an 8-fold enhancement compared to S0. Experimental investigations [19] show that the superlinear dependence of the FSP fluorescence is tightly linked to stimulated ZnO plasma emission (*i.e.*, *N*-line), proving the occurrence of a radiative energy transfer from the ZnO thin film towards the FSP layer.

**Figure 5:**
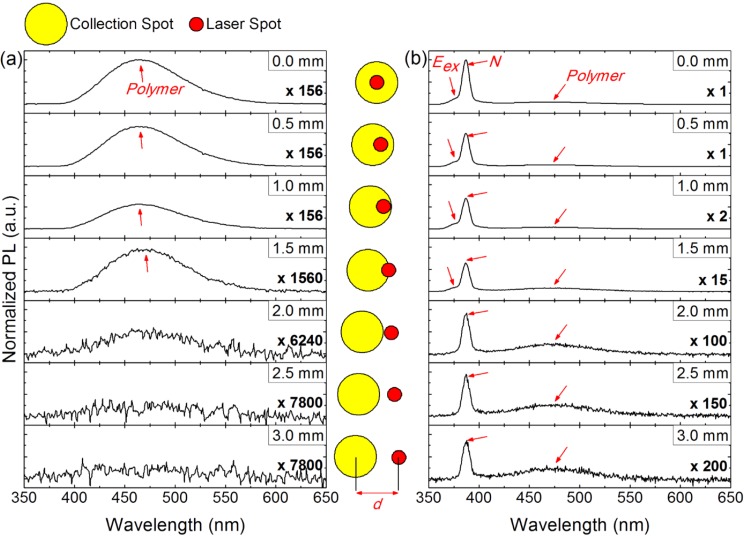
Evolution of the photoluminescence (PL) spectra for the polymer fluorescent film coated on quartz, S0 sample (**a**) and on the ZnO layer, S1 sample (**b**) as a function of the distance (*d*) separating the center laser spot and the axis of the collection objective. The PL spectra are all plotted on the same *y*-axis scale and normalized by the PL intensity recorded at the maximum emission wavelength of the *N*-line for *d* = 0 mm. The inset between the two figures presents a sketch of the evolution of d. Reprinted with permission from [19]. Copyright 2014 American Chemical Society.

The fluorescence enhancement, in the case of S1, is attributed to an efficient excitation of the polymer layer lying outside of the area defined by the laser spot via the waveguided ZnO stimulated emission (*i.e.*, a “geometrical” effect). The geometrical effect is confirmed by photoluminescence (PL) measurements shown in Figure 5.

Figure 5 shows the evolution of the PL spectra collected on S0 (a) and S1 (b) as the misalignment (*d*) between the collection axis and the laser spot is gradually increased. The value of *d* is indicated in the legend of each graph and is illustrated in the inset of Figure 5. The PL setup, considered here, has a collection spot area diameter of 3 mm and a laser spot diameter of 1mm. At *d* = 2 mm, the collection spot is thus externally tangent to the laser spot, as illustrated in Figure 5.

Figure 5a shows that, in the case of S0, the fluorescent polymer area is solely defined by the laser spot. In fact, the polymer fluorescence spectrum, as shown in Figure 5a, totally vanishes for *d* ≥ 2 mm. For S0, the polymer layer is uniquely excited by the laser beam. On the other hand, Figure 5b clearly shows that, in the case of S1, the fluorescent polymer area is strictly larger than that of the laser spot. For S1, the polymer fluorescence spectrum, as shown in Figure 5b, is still collected for *d* ≥ 2 mm. The polymer fluorescence, in the case of S1, results of two fluorescing areas. A smaller area, defined by the laser spot (depicted by the dark green star in Figure 1b), that is mainly excited by the laser beam; and a larger area, defined outside of the laser spot (depicted by the light green in Figure 1b), that is mainly excited by the waveguided ZnO stimulated plasma *N*-line emission at 386 nm. This is evidenced by the *N*-line peak collected at *d* ≥ 2 mm. Meanwhile, the spectral filtering (*i.e.*, attenuation) for *d* ≥ 2 mm of the ZnO spontaneous emission at 375 nm, denoted by *E*_ex_, evidences on the waveguided nature of the *N*-line (the extinction coefficient κ of ZnO is decreased from 0.311 at 375 nm to 0.056 at 386 nm).

The experimental investigation reveal almost one order of magnitude fluorescence enhancement by spincoating the FSP on a ZnO thin film. Furthermore, the experimental investigation proves the important role of waveguided ZnO emission in having an efficient fluorescence excitation of extremely thin FSP layers. The experimental results are in good agreement with the previously discussed theoretical results which also revealed the importance of waveguided ZnO emission. However, the theoretical modeling predicted a 30-fold enhancement of the polymer fluorescence. The prediction is overestimated mainly due to the fact that the theoretical modeling did not account for the real values of the polymer and ZnO quantum yield.

As previously mentioned, nanostructured materials present many interesting properties which can be extremely beneficial for sensing applications. In the following, we present a preliminary theoretical study on the enlarged sensing surface and photonic band-gap of ZnO nanostructures. The theoretical study, presented henceforth, considers the case of hexagonally distributed ZnO nanorods and perforated ZnO thin films. Hexagonal distribution is chosen as it presents the most compact packing arrangement and therefore the most interesting geometric and photonic properties.

Nanostructured materials can exhibit extremely high specific surfaces which can help increase polymer sensitivity by enlarging the polymer sensing surface and enhancing fluorescence signal without diminishing their response time and quenching efficiency [16]. We define the gain in sensing surface (*R_G_*) as the ratio between the surface area of a FSP layer coated on a nanostructured support and that of a FSP layer coated on a smooth plan. Considering a homogeneous FSP coating over the whole coated area, *R_G_*, of any given geometry, can be determined through simple geometrical calculations.

**Figure 6:**
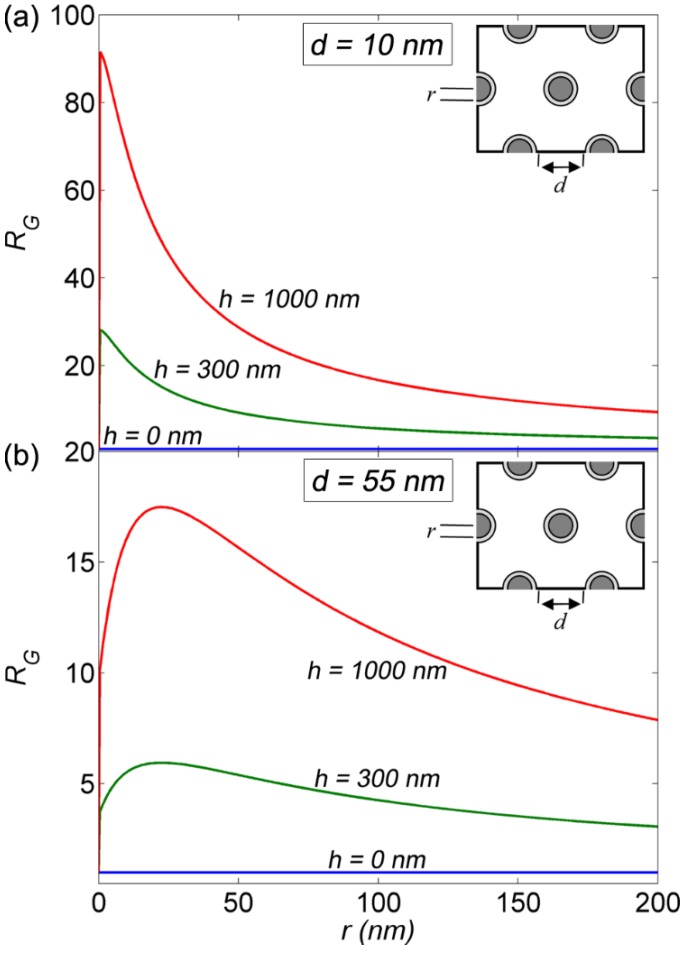
Evolution of the surface gain (*R*_G_) for hexagonally ordered ZnO nanorods as a function of the radius (*r*) and for various nanorod height (*h*). (**a**) presents the case of *d* = 10 nm and (**b**) presents the case of *d* = 55 nm.

Figure 6 represents the evolution of *R*_G_ for a hexagonal array of nanorods as a function of the rods radius (*r*) for various rod heights (*h* = 0 nm, 300 nm, and 1000 nm) and for two fixed values of the air gap (*d* = 10 nm (a) and 55 nm (b)) separating two adjacent polymer coated nanorods (see Figure 6 inset). For the figure insets hereafter grey is for ZnO and white is for air. The calculated values of *R_G_* are obtained for a fixed polymer thickness of 5 nm. As shown in Figure 6a,b, for *h* = 0 or *r* = 0 (*i.e.*, no nanorods), the surface presents no gain and, as expected, *R_G_* has a value of 1. However, for any given value of *h* and *r* differing from 0, *R*_G_ presents a value higher than 1. In other words, any array of nanorods, no matter the structure parameters, will exhibit an enlarged sensing surface compared to a smooth plane. In general, Figure 6 shows that the enlarged sensing surface (*i.e.*, *R*_G_) is more important for a higher (*i.e.*, increasing *h*) and denser (*i.e.*, decreasing *d*) stacking of nanorods. For *h* > 0, *R*_G_ exhibits an optimum value. As seen in Figure 6a,b, the optimum value of *R*_G_ shifts towards higher values of *r* as *d* increases. For the considered structure parameters, the highest calculated value for *R*_G_ reaches a value of ~90 (almost two order of magnitude) and is obtained for *r* ≈ 0.4 nm, *d* = 10 nm and *h* = 1000 nm. For *d* = 10 nm (Figure 6a), the optimum value of *R*_G_ exists at the smallest value of *r* ≠ 0, which results in a very abrupt change from *R_G_* = 1 at *r* = 0 to *R_G_* ≈ 90 at *r* ≈ 0.4. Nonetheless, nanorods of such high aspect ratio (*r* ≈ 0.4 nm and *h* = 1000 nm) are experimentally hard to realize. More reasonable structure parameters, such as *r* = 50 nm, *d* = 55 nm and *h* = 1000 nm, reduces the surface gain to an order of a 15-fold (one order of magnitude). Nonetheless, this remains an important enhancement factor. It is essential to stress that a facile technique for the growth of urchin-like ZnO nanostructures was recently reported by our team [35]. The grown urchin-like structures exhibited a high aspect ratio with ZnO nanorods of a diameter of around 15 nm and a length of 500 nm. With such dimensions, the urchin-like nanostructures should result in a gain in sensing surface of more than two-orders of magnitude as compared to a flat surface. Moreover, the urchin-like nanostructures exhibited a high internal quantum efficiency of 23% at room temperature. The luminescence properties of urchin-like nanostructures are thus also potentially interesting for achieving an efficient G-WET.

**Figure 7:**
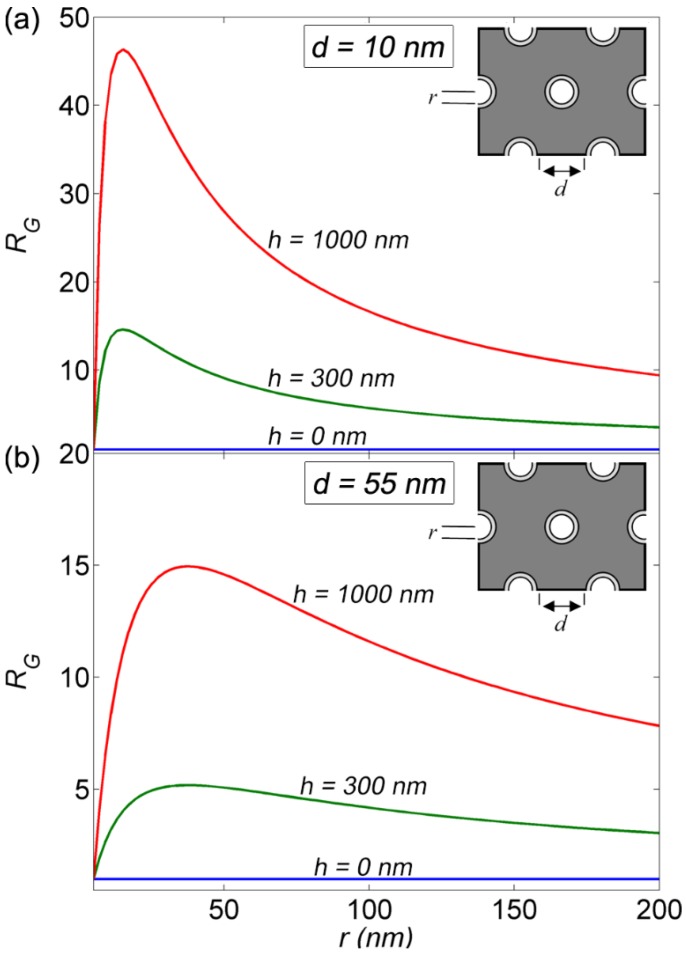
Evolution of the surface gain (*R*_G_) for hexagonally ordered nanoholes as a function of the radius (*r*) and for various nanorod heights (*h*). (**a**) presents the case of *d* = 10 nm and (**b**) presents the case of *d* = 55 nm.

In analogy to Figure 6, Figure 7 represents the evolution of *R_G_* for a hexagonal array of nanoholes, perforated in a ZnO thin film, as a function of the nanoholes radius *r* for various values of film thicknesses (*h* = 0 nm, 300 nm, and 1000 nm) and for two fixed values of the air gap (*d* = 10 (a) and 55 nm (b)) separating two adjacent polymer coated nanoholes (see Figure 7 inset). The polymer thickness is again fixed to 5 nm. The calculated values of *R_G_* for the nanoholes, shown in Figure 7, exhibit a similar behavior to the calculated values of *R_G_* for the nanorods, shown in Figure 6. As seen in Figure 7, gains in sensing surface are expected independently of structures parameters (*i.e.*, *R*_G_ is bigger than 1 for any value of *r* and *h* differing from 0). Moreover, the enlarged sensing surface (*i.e.*, *R*_G_) is more important for a deeper (*i.e.*, increasing *h*) and denser (*i.e.*, decreasing *d*) stacking of nanoholes. However, perforated thin films, in general, present smaller values of *R*_G_ compared to nanorods. The highest *R_G_* calculated for perforated thin films reaches a value of ~45 for *r* ≈ 15 nm, *d* = 10 nm and *h* = 1000 nm. However, arrays of nanoholes with such dimensions are hard to perforate, especially in ZnO thin films. Moreover, nanoholes with such radius (*r* ≈ 15) are hard to homogeneously coat with a polymer, due to the limited polymer penetration inside the holes (surface wetting problem). Again, more feasible nanoholes [20] lead to a 15-fold enhancement (one order of magnitude) in the sensing surface.

As previously mentioned, optical gain is an essential feature of the G-WET concept. It is clear that higher optical gains and reduced lasing thresholds are desirable for more efficient energy transfers. ZnO micro- and nanostructures can be thus important for such purposes. We recently showed [21] that ZnO microdisks exhibited a reduced lasing threshold compared to the initial lasing threshold measured for the ZnO thin film before microstructuring. The reduced lasing threshold was attributed to the optical feedback occurring inside the microstructures. Such structures are extremely interesting as they are easy to realize, especially at a large scale, and they should allow for an efficient G-WET process at low pumping intensities. On the other hand, templated growth offers an easy and cheap alternative for the realization of nanostructures. Recent advances on assisted sphere self-assembly in our team allow for the realization of hexagonally ordered ZnO nanostructures on a large scale [36]. Such large scale and cheap ZnO nanostructuring is interesting for many functional applications including sensors.

**Figure 8:**
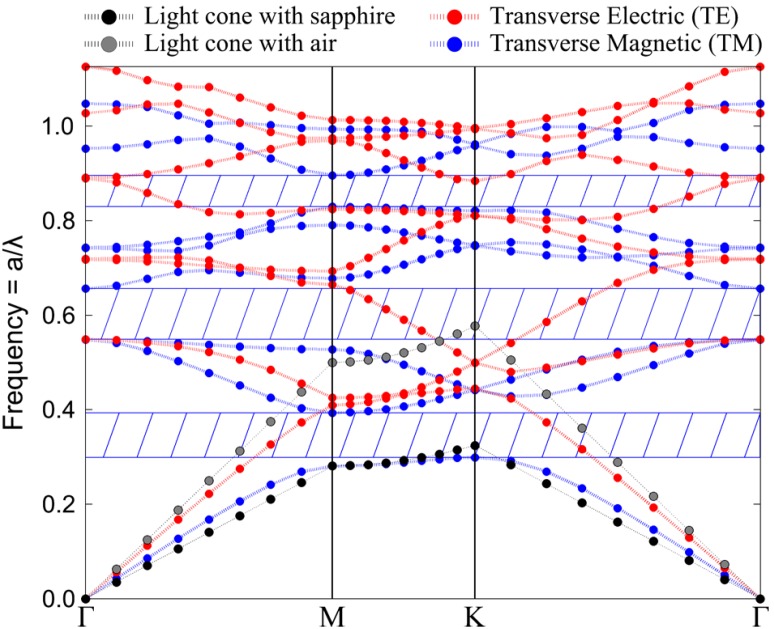
Photonic band diagram of ZnO nanorods having radius to center-center periodicity ratio of 0.3.

Hexagonal arrays of ZnO nanostructures are photonic crystals (PhCs) and thus present interesting photonic properties. Recent studies have shown that the photonic band-gap (PBG) plays a crucial role in reducing the lasing threshold in ZnO PhCs [20]. Therefore, it is essential to determine the structure parameters that are required to obtain the desired PBG. For this purpose, plane wave expansion (PWE) calculations [37,38] were carried out using a photonics design automation software ‘RSoft’. PWE method offers a fast and accurate technique to determine the photonic band diagram, and therefore the PBG, of PhCs. Before proceeding, it is essential to note that, hereafter, transverse magnetic (TM) polarization is defined as the electric field being parallel to the rods/holes; while transverse electric (TE) polarization is define as the electric field running around the rods/holes. Figure 8 shows the photonics band diagram obtained at λ = 375 nm for a hexagonal distribution of ZnO nanorods having radius on period ratio (*r*/*a*) of 0.3. The *x*-axis represents the wave-vector *k* (*k_x_*, *k_y_*), where Γ (0,0), M (π/*a*, $-\pi /\sqrt{3}a$), and K (4π/3*a*, 0) are the symmetry points of the hexagonal lattice in reciprocal space. Figure 8 shows that the considered ZnO nanorods structure parameters exhibit 3 TM-polarized PBGs. It is important to note that the photonic band diagram of a given periodic geometry is solely dependent on *r*/*a*. By plotting the evolution of the PBG as a function of *r*/*a*, we obtain the photonic band-gap map. Moreover, the *y*-axis is conventionally expressed in normalized frequency which is equivalent to *a*/λ; such is the case in Figure 8. However, the *y*-axis hereafter will be directly expressed in terms of the center-center periodicity (*a*) for more practical reasons.

**Figure 9:**
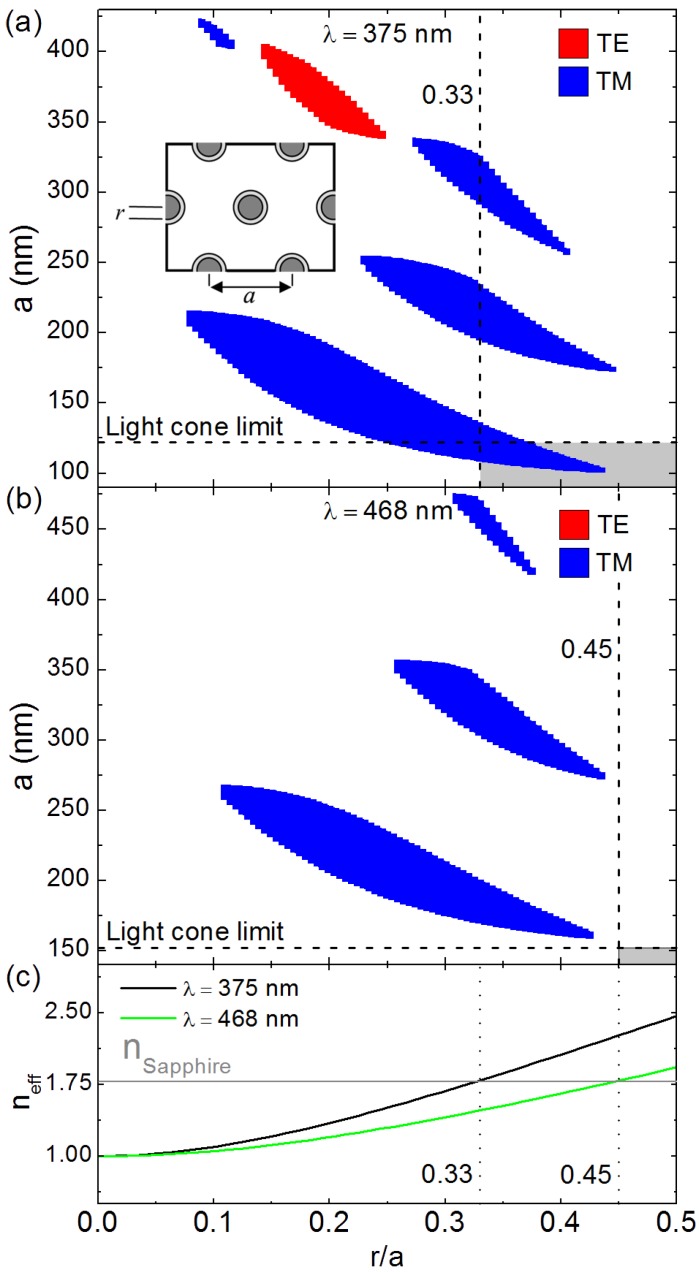
(**a**) Photonic band-gap map of ZnO nanorods for λ = 375 nm; (**b**) Photonic band-gap map of ZnO nanorods for λ = 468 nm; (**c**) Effective refractive index of the ZnO nanorods obtained by Maxwell-Garnett approximation for λ = 375 nm and 468 nm.

Figure 9a,b respectively show the PBG map calculated for a hexagonal lattice of ZnO nanorods at λ = 375 nm (ZnO excitonic emission) and λ = 468 nm (polymer emission) for the TE (red) and TM (blue) modes. As expected, Figure 9a,b shows that ZnO nanorods have a preferably TM polarized band-gap. For λ = 375 nm, Figure 9a shows the existence of several TM band-gap for *r*/*a* values ranging from 0.08 to 0.43 and a values ranging from 100 nm to 430 nm. In addition, Figure 9a shows the existence of a TE bandgap for *r*/*a* values ranging from 0.15 to 0.25 and a values ranging from 337 nm to 404 nm. On the other hand, for λ = 468 nm, Figure 9b shows only TM polarized bandgaps for *r*/*a* values ranging from 0.11 to 0.43 and a values ranging from 160 nm to 470 nm. The photonic gap map is an effective tool for the choice of the structure parameters *a* and *r*. However, other considerations must also be taken into account in order to make the best choice of structure parameters. For instance, photonic band-gaps only affect light that is guided within the structured layer (*i.e.*, ZnO nanorods). We recall that here we are dealing with ZnO nanostructures on sapphire. For such nanostructures, PBGs are only interesting if they lie under the limit *a*/λ = 0.325 defined by the light cone between the ZnO structures and the sapphire substrate [34]. For structures having *a*/λ = 0.325, light begins leaking into the sapphire substrate. Therefore, structures with *a* < 122 nm for λ = 375 nm and *a* < 152 nm for λ = 468 nm would be most suitable for having a photonic band-gap effect. Another important issue is the effective refractive index. The nanostructured layer presents effective medium resulting from the mixture of air and ZnO. It is, therefore, essential for the nanostructured layer to have an effective refractive index greater than that of the sapphire substrate in order to support guided modes. Figure 9c presents the evolution of the effective refractive index (*n*_eff_) obtained using Maxwell-Garnett approximation [39,40] for a hexagonal lattice of ZnO nanorods as a function of *r*/*a*. As expected, Figure 9c shows that *n*_eff_ increases with increasing *r*/*a*. Moreover, Figure 9c shows that *n*_eff_ is only greater than the refractive index of sapphire for *r*/*a* > 0.33 for λ = 375 nm and *r*/*a* > 0.45 for λ = 468 nm. Figure 9a thus shows that the limits of the light cone and the effective refractive index at λ = 375 nm (*a* < 122 nm and *r*/*a* > 0.33) are only verified by a narrow window of the first order TM-polarized PBG. Meanwhile, Figure 9b shows no PBG verifying the light cone and effective refractive index limits at λ = 468 nm (*a* < 152 nm and *r*/*a* > 0.45). It is important to stress that ZnO emission is mostly TE polarized [33,34]. Thus, for a PBG effect, perforated ZnO photonic crystal slabs would be more suitable than ZnO nanorods, as they present preferably TE polarized bandgaps [20,34]. Nevertheless, ZnO nanorods are more interesting for having high light extraction efficiency, due to their low effective refractive index. Moreover, ZnO nanorods would be more interesting for enlarging the sensing surface. ZnO nanorods present a more important specific surface (Figure 6) compared to perforated ZnO thin films (Figure 7).

**Figure 10:**
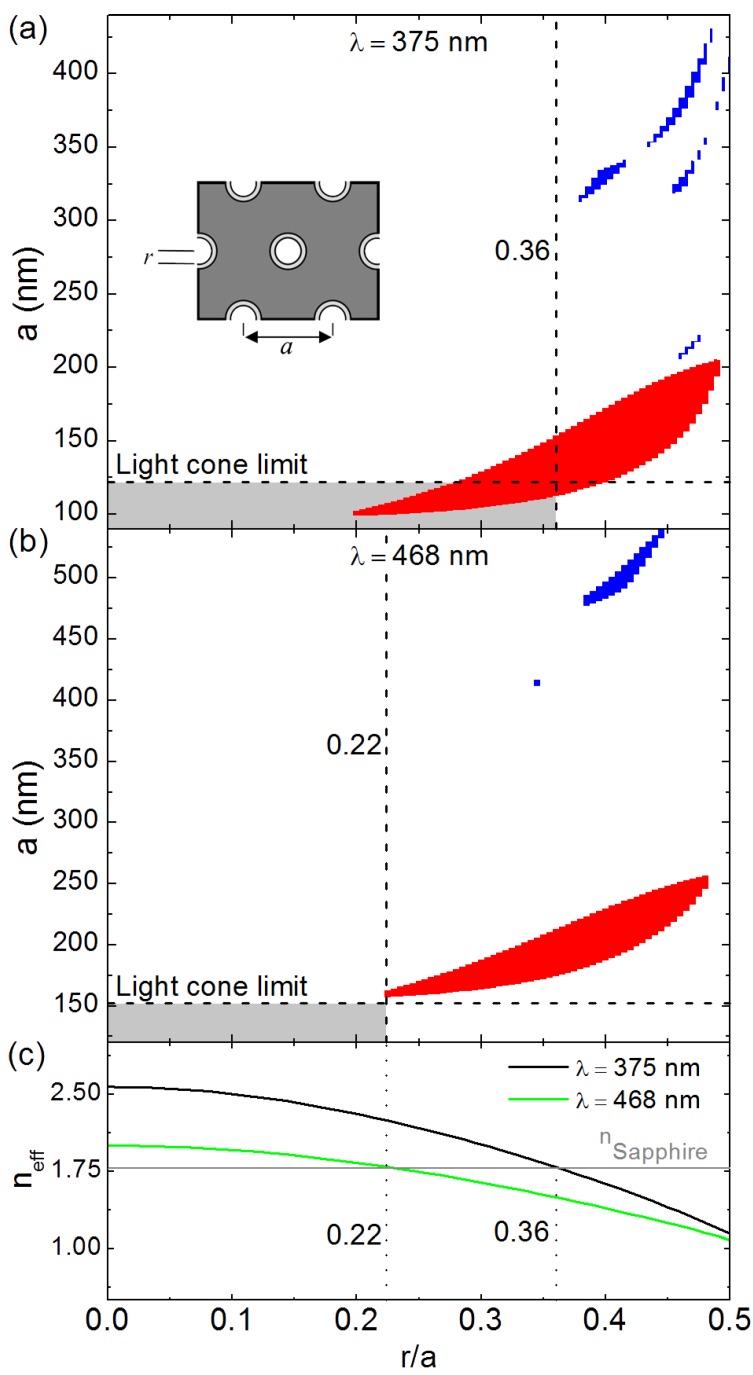
(**a**) Photonic band-gap map of perforated ZnO slab for λ = 375 nm; (**b**) Photonic band-gap map of perforated ZnO slab for λ = 468 nm; (**c**) Effective refractive index of the perforated ZnO slab obtained by Maxwell-Garnett approximation for λ = 375 nm and 468 nm.

Similarly to Figure 9, Figure 10a,b respectively show the PBG map calculated for a hexagonal lattice of air nanoholes perforated in a ZnO slab at λ = 375 nm (ZnO excitonic emission) and λ = 468 nm (polymer emission) for the TE (red) and TM (blue) modes. As expected, Figure 10a,b shows that the perforated ZnO slab has a preferably TE polarized band-gap. For λ = 375 nm, Figure 10a shows the existence of TE band-gap for *r*/*a* values ranging from 0.2 to 0.49 and *a* values ranging from 100 nm to 204 nm. In addition, Figure 10 shows the existence of several TM bandgaps for *r*/*a* values ranging from 0.38 to 0.5 and *a* values ranging from 200 nm to 430 nm. On the other hand, for λ = 468 nm, Figure 10b shows TE polarized bandgap for *r*/*a* values ranging from 0.22 to 0.48 and *a* values ranging from 160 nm to 256 nm. Figure 10b also shows the existence of a TM polarized bandgap for *r*/*a* values ranging from 0.34 to 0.44 and *a* values ranging from 415 nm to 540 nm. As previously mentioned, perforated ZnO slabs are most suitable for a pronounced photonic band gap effect on ZnO emission. We remind that *a* < 122 nm for λ = 375 nm and *a* < 152 nm for λ = 468 nm define the limits of the light cone between the ZnO slab and the sapphire substrate. Figure 10c presents the evolution of the effective refractive index (*n*_eff_) obtained using Maxwell-Garnett approximation for a hexagonal lattice of air nanoholes perforated in ZnO slab as a function of *r*/*a*. As expected, Figure 10c shows that *n*_eff_ decreases with increasing *r*/*a*. Figure 10c shows that *n_eff_* is only greater than the refractive index of sapphire for *r*/*a* < 0.36 for λ = 375 nm and *r*/*a* < 0.25 for λ = 468 nm. Figure 10a thus shows that the limits of the light cone and the effective refractive index at λ = 375 nm (*a* < 122 nm and *r*/*a* < 0.36) are only verified by a narrow window of the first order TE-polarized PBG. Meanwhile, Figure 10b shows no PBG verifying the light cone and effective refractive index limits at λ = 468 nm (*a* < 152 nm and *r*/*a* < 0.25). Structures which have *r*/*a* = 0.3 and *a* = 116 nm should present interesting photonic band-gap properties.

Photonic band-gaps result from an ordered optical feedback occurring over a large scale. However, ZnO nanostructures, such as nanorods, can offer many photonic properties over a much smaller, even individual, scale. Such photonic properties can be extremely interesting for the fluorescence excitation of extremely thin polymer layers.

**Figure 11:**
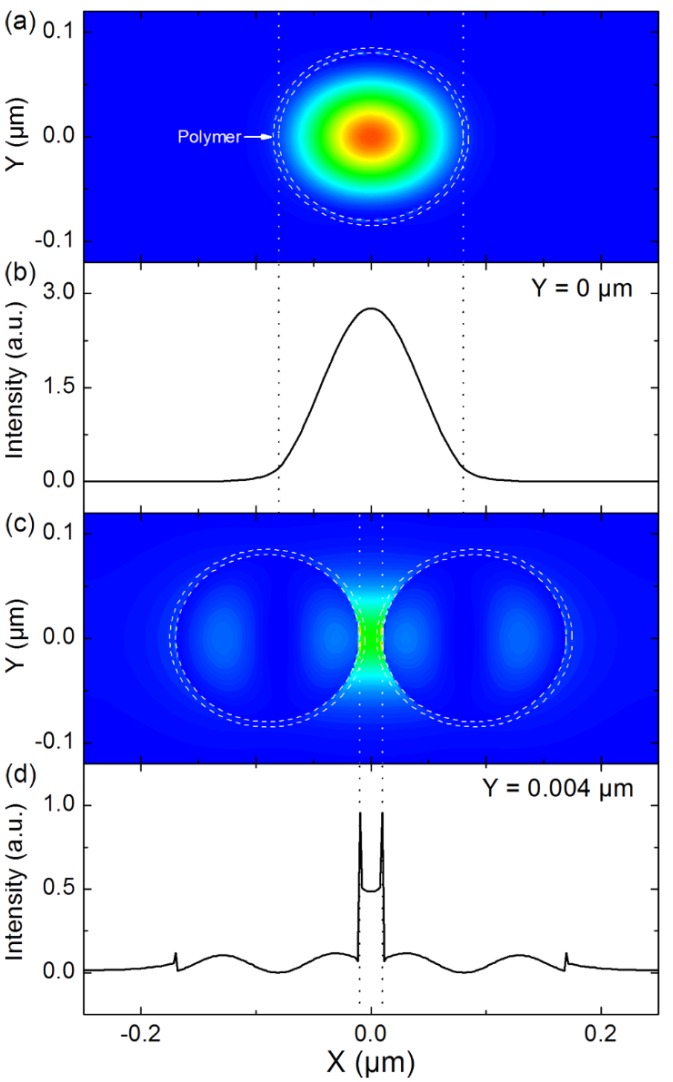
(**a**) Contour map of the Poynting vector amplitude distribution for the guided mode in a ZnO nanorod (defined by the inner dashed circle) with a diameter of 160 nm; (**b**) X-cut at *Y* = 0 µm for the guided mode profile for the ZnO nanorod; (**c**) Contour map of the Poyting vector amplitude distribution for the gap mode for 2 coupled ZnO nanorods (defined by the inner dashed circles). The coupled nanorods have a diameter of 160 nm and are separated by a 20 nm air gap; (**d**) X-cut at *Y* = 0.004 µm for the gap mode profile for the coupled ZnO nanorods.

Figure 11 shows finite element method (FEM) results for a ZnO nanorod (Figure 11a,b) and two coupled ZnO nanorods (Figure 11c,d). The FEM calculations were realized at λ = 375 nm. For the two structures, the ZnO nanorods has a diameter of 160 nm. The two coupled ZnO nanorods are separated by a 20 nm air gap. Figure 11a shows the Poynting vector amplitude distribution of the guided mode in the ZnO nanorod. As seen is Figure 11a, a ZnO nanorod can support guided modes even at a nanometric scale. Figure 11b shows an X-Cut of the guided mode profile at the middle of the ZnO nanorod (*i.e.*, *Y* = 0 µm). As expected, Figure 11b shows that the guided mode has a Gaussian profile. The guided mode is highly confined inside the ZnO nanorod. Only a small part of the evanescent tail, sticks out of the ZnO nanorod. Figure 11c shows the Poynting vector amplitude distribution of the gap mode for the coupled ZnO nanorods. As seen in Figure 11c, the energy of the gap mode is confined within the 20 nm air gap in between the two ZnO nanorods. Figure 11d shows a X-cut of the gap mode profile slight above the middle of the two nanorods (at *Y* = 0.004 µm). Figure 11d reveals the existence of a sharp peak at the edge of each ZnO nanorod. Figure 11d thus shows that the energy of the gap mode is strongly confined at the air/ZnO interface. The dashed circles in Figure 11a,c defines the limits of the area that would occupy a 5 nm thick polymer coated on the ZnO nanorods. For the polymer fluorescence excitation, two quantities are to be considered, the excitation intensity over the whole polymer area and the excitation intensity at a local scale. The excitation intensity over the whole polymer area for Figure 11c (*i.e.*, the coupled ZnO nanorods) is almost 2 times higher than that of Figure 11a (*i.e.*, the single ZnO nanorod). The gap mode can thus lead to a two-fold enhancement in the polymer fluorescence. Compared to the guided mode (Figure 11a), the gap mode (Figure 11c) is confinement within a much smaller surface. The local excitation intensity of the gap mode in the polymer layer (Figure 11d) can reach up to 4–5 times that of the guided mode. Such high local intensities can be extremely beneficial for polymer layers exhibiting ASE, as they can lead to a drastic decrease of the lasing threshold. Such gap modes can be manifested in densely stacked randomly ordered ZnO nanorods.

To summarize, nanostructured ZnO offers a multitude of possible choices to enhance the signal of nanothin fluorescence sensing polymers. Random arrays of ZnO nanostructures, such as densely stacked ZnO nanowires with high aspect ratio, have a very large sensing surface and can exhibit strongly confined gap modes within the polymer volume, which can lead to a drastic enhancement in polymer fluorescence. Ordered arrays of ZnO nanostructures, on the other hand, offer the advantage of having a photonic bandgap. The photonic bandgap of perforated ZnO thin films is most appropriate for such purposes, as ZnO emission is preferable TE polarized. Perforated ZnO thin films exhibit lower lasing threshold, which can lead to a more efficient G-WET effect and therefore a better fluorescence excitation. It is evident that the use of ZnO nanostructures can most certainly lead to a drastic enhancement in the polymer fluorescence. However, at this level, it is hard to estimate, the enhancement factors that the various ZnO nanostructures would present, as the behavior of the polymer fluorescence towards the various photonic phenomena in act is still not deeply understood. Both experimental and theoretical (such as active FDTD) investigations are thus needed in order to determine the enhancement factors and to further understand the impact that each effect has on the polymer fluorescence.

## 4. Conclusions

In conclusion, we introduced a new photonic concept, called G-WET, to enhance the fluorescence of extremely thin FSP layers. The G-WET concept proposes the use of optically active material for an efficient polymer fluorescence excitation. In a primary approach, the G-WET concept is theoretically and experimentally validated for the case of an extremely thin FSP layer spincoated on a ZnO thin film. The G-WET concept is demonstrated to induce an 8-fold increase in the polymer fluorescence. The photonic concept is afterwards extended to nanostructured materials. A theoretical study on the enlarged sensing surface and photonic bandgap of hexagonally organized ZnO nanorods and perforated ZnO thin films is presented. The theoretical study presents preliminary results showing the importance of nanostructured active materials in an ultimate approach to enhance the FSP fluorescence.
